# Rainfall pulse response of carbon fluxes in a temperate grass ecosystem in the semiarid Loess Plateau

**DOI:** 10.1002/ece3.4587

**Published:** 2018-10-23

**Authors:** Yakun Tang, Jun Jiang, Chen Chen, Yunming Chen, Xu Wu

**Affiliations:** ^1^ State Key Laboratory of Soil Erosion and Dryland Farming on the Loess Plateau Institute of Soil and Water Conservation Northwest A&F University Yangling China; ^2^ State Key Laboratory of Soil Erosion and Dry‐land Farming on the Loess Plateau Institute of Soil and Water Conservation Chinese Academy of Sciences and the Ministry of Water Resources Yangling China; ^3^ University of Chinese Academy of Science Beijing China

**Keywords:** carbon fluxes, eddy covariance, loess Plateau, rainfall pulse, semiarid region

## Abstract

Rainfall pulses can significantly influence carbon cycling in water‐limited ecosystems. The magnitude of carbon flux component responses to precipitation may vary depending on precipitation amount and antecedent soil moisture, associated with nonlinear responses of plants and soil microbes. The present study was carried out in a temperate grass ecosystem during 2013–2015 in the semiarid Loess Plateau of China, to examine the response of carbon fluxes to precipitation using the “threshold‐delay” model. The unique contribution of environmental variables such as precipitation amount and antecedent soil moisture before rainfall (SWC_antecedent) to carbon fluxes in response to rainfall was also investigated. The lower threshold of effective rainfall was 6.6 mm for gross ecosystem production (GEP), 8.5 mm for net ecosystem production (NEP), and 4.5 mm for ecosystem respiration (RE); and the upper threshold of effective rainfall was 21.4 mm for GEP and NEP, and 16.8 mm for RE. Rainfall amount was positively affected the relative rainfall responses of GEP, NEP, and RE. However, SWC_antecedent at 20 cm soil depth offset the response of GEP to rainfall pulses, and SWC_antecedent at 5 cm soil depth offset the response of NEP and RE to rainfall pulses, with corresponding partial slopes of linear regressions of −0.50, −0.40, and −0.52. These results indicated that NEP was more sensitive to rainfall pulses and RE was more sensitive to SWC_antecedent. These results demonstrate the importance of rainfall events of <10 mm and that the negative effect of SWC_antecedent should also be considered when estimating ecosystem carbon fluxes in this semiarid region.

## 1 INTRODUCTION

Precipitation in arid and semiarid ecosystems is highly variable and discontinuous, with periods of low water availability separated by episodic events (Zeppel, Macinnis‐Ng, Ford, & Eamus, 2008; Zhao & Liu, 2010). The temporal variability of rainfall pulses, associated with the nonlinear responses of plants and soil microbes, constrains ecosystem carbon processes through the influence on water availability (Huxman et al., 2004; Tang et al., 2016). Climate change models indicate that the dynamics and distribution of rainfall will become more variable and exhibit greater influence on ecosystem carbon dynamics than other environmental variables (e.g., CO_2_ concentration and temperature), although these models differ with changes in precipitation amount (Hao et al., 2013; Ryan et al., 2017). Understanding the responses of carbon fluxes to rainfall pulses is essential for comprehending the carbon cycles in the context of climate change, especially in water‐limited ecosystems (Peng et al., 2013; Tang et al., 2016).

Semiarid grassland ecosystems occupy approximately 9.1 × 10^6^ km^2^, or 20% of the earth's land surface area, and provide valuable CO_2_ sequestration, calculated as net ecosystem production (NEP) (Liu et al., 2017; Scott, Hamerlynck, Jenerette, Moran, & Barron‐Gafford, 2010). Precipitation effects on NEP depend on differential responses of gross ecosystem production (GEP) and ecosystem respiration (RE). Moreover, different responses of carbon fluxes to rainfall pulses can be attributed to differing metabolic responses of plant and soil microbes (Hao et al., 2010; Potts et al., 2006). Generally, increased rainfall enhances GEP more than RE and results in increased net carbon uptake (Chen et al., 2015; Guo et al., 2016; Huxman et al., 2004). However, Austin et al. (2004) and Kurc and Small (2007) indicated that RE in grass ecosystems was more sensitive to small rainfall amounts, and GEP was more sensitive to larger amounts. In addition, precipitation manipulation experiments suggested that the effect of small rainfall events (e.g., <5 mm) on carbon fluxes in arid and semiarid regions was more essential than the total rainfall amount (Knapp et al., 2002; Suseela, Conant, Wallenstein, & Dukes, 2012). Until recently, the threshold‐delay (Ogle & Reynolds, 2004), pulse‐reserve (Noy‐Meir, 1973), and two‐layer hypothesis models (Walter, 1971) have been used to analyze rainfall responses of plant species or ecosystems. The threshold‐delay model has been widely use in shrub (Jian, Wu, Hu, & Zhang, 2016; Zhao & Liu, 2010) and grass (Zeppel et al., 2008) ecosystems to identify the lower effective rainfall amount that affects vegetation.

The effect of rainfall on carbon fluxes may also depend on soil water conditions (Huxman et al., 2004; Zeppel et al., 2008). For example, Potts et al. (2006) suggested that antecedent soil moisture before rainfall (SWC_antecedent) significantly affects the variation carbon fluxes in a bunchgrass ecosystem. Furthermore, Hao et al. (2010) also indicated that high SWC_antecedent can offset the sensitivity of GEP to rainfall pulses in a semiarid grass ecosystem. However, the relative importance of rainfall amount and SWC_antecedent to the responses of carbon fluxes to rainfall pulses is poorly documented (Hao et al., 2010; Tang, Wen, Sun, Zhang, & Wang, 2014). Accurate quantification of the unique contribution of these factors is needed to estimate the response of carbon flux to rainfall pulses.

To overcome habitat degradation and soil erosion in the Chinese Loess Plateau, the “Grain for Green” project was launched in the last decade, and grassland now occupies more than 39.2% of the region's total vegetation area (Liu et al., 2017). The GEP and NEP are considered to be direct indicators of the growth conditions of grass ecosystems (Guo et al., 2016; Scott et al., 2010). Similar to other semiarid grass ecosystems (Hao et al., 2013; Ryan et al., 2017), the carbon fluxes in this region are likely to be sensitive to rainfall pulses. In the present study, 3 years (2013–2015) of carbon fluxes were examined in a grass ecosystem in the semiarid Loess Plateau. The objectives were to (a) determine the rainfall threshold that significantly increases GEP, NEP, and RE, and (b) identify the unique contribution of rainfall amount and SWC_antecedent to these carbon fluxes in response to rainfall.

## 2 MATERIALS AND METHODS

### 2.1 Study site

The Ansai flux observation site (36.85°N, 109.32°E, and elevation 1,260 m) belongs to the Chinese Ecosystem Research Network (CERN) in the middle of the Loess Plateau and is part of ChinaFLUX. The total and core experimental area occupies 2.56 and 0.96 ha, respectively, with the corresponding slopes within 8–12.5° and 3.5–8.5°. The climate is temperate semiarid, and the annual average (±*SD*) rainfall and air temperature were 491.5 ± 120.2 mm and 10.3 ± 0.4°C (1988–2012), respectively. In this region, the loess is characterized as silt loam with a depth more than 50 m. The bulk density was 1.20 ± 0.03 and 1.29 ± 0.05 g/cm^3^ at soil depths 0–5 and 5–20 cm, respectively, and corresponding total porosity was 52.14 ± 1.13 and 49.66 ± 0.98% based on a survey carried out in July 2015.

The observation area has been protected since 2002 and is dominated by natural secondary grass ecosystem, comprising *Artemisia scoparia* and *Glycyrrhiza uralensis*. The mean height and density for *A. scoparia* were 0.53 m and 46 plants/m^2^, respectively, and correspondingly for *G. uralensis* were 0.25 m and 26 plants/m^2^, according to a survey carried out in July 2015. The coverage of this grass community ranged within 85%–92% during a survey conducted during May and September 2015.

### 2.2 Investigation of root systems

In July 2014, 20 individual plants of *G. uralensis* and *A. scoparia* of approximately average height were selected for whole plant root excavation. A spade was used to dig up the roots at depths of 0–10, 10–20, 20–30, and 30–50 cm. Then, the fine root (root diameter <2 mm) surface area of each grass species was calculated for each depth interval using WinRHIZO (Regent Instruments Inc., Quebec, Canada). In addition, the surface area of dominant grass species was also calculated using the mean density of *A. scoparia* (46 plants/m^2^) and *G. uralensis* (26 plants/m^2^).

### 2.3 Eddy covariance and environmental variable measurements

Since August 2012 at the center of core experimental area, the CO_2_ fluxes were detected using an CO_2_/H_2_O analyzer (LI‐7500, Li‐Cor Inc., Lincoln, NE, USA) mounted at 2 m above the soil surface, and a 3D sonic anemometer (CSAT3, Campbell Scientific Inc., Logan, UT, USA). A CR3000 datalogger (Campbell Scientific Inc.) was used to collect and record 10‐Hz and half‐hour mean fluxes data.

The photosynthetically active radiation was measured using a LI190SB (Licor Inc.), and net radiation (*R*_n_) was determined using a CNR‐1 (Kipp & Zonen Inc., The Netherlands). A HMP45C (Campbell Scientific Inc.) was used to detect air relative humidity and temperature. A 52203 rain gauge (RM Young Inc., USA) was used to determine rainfall. Soil volumetric water moisture (SWC) and temperature at one soil profile (5, 20, and 50 cm depths) were recorded with CS616‐L TDR probes (Campbell Scientific Inc.) and 105T thermocouples (Campbell Scientific Inc.), respectively. Three HFT‐3 plates (Campbell Scientific Inc.) at 5 cm soil depth were used to detect soil heat flux (G). The half‐hour values of all above variables were stored by CR1000 dataloggers (Campbell Scientific Inc.).

### 2.4 Processing of eddy covariance

The effect of air flow irregularity or instrument tilt for half‐hour fluxes was removed by planar fit rotation (Wilczak, Oncley, & Stage, 2001). Anomalous flux values caused by rainfall were eliminated. Based on the friction velocity (u*) effect identified by Reichstein et al. (2005), the fluxes were removed when u* was <0.21 m/s to obtain reliable night fluxes (solar elevation angle <0). During May–September, the average reliable daytime and nighttime carbon flux data coverage for 2012–2014 was 83.2 ± 7.6% and 35 ± 4.1%, respectively. The quality of the eddy covariance system was assessed by the energy balance ratio (EBR), calculated as (LE + *H*)/(*R*_n_ − *G*) (Tang et al., 2016; Wilson et al., 2002). LE is the latent heat flux (W/m^2^), and *H* is the sensible heat flux (W/m^2^). The average EBR was 0.81 ± 0.11 among 2013–2015, which was larger than the average EBR (0.79) of FLUXNET sites (Wilson et al., 2002).

Linear interpolation was used to fill carbon and meteorological gaps that <2 hr, when the continuous 2 hr data before and after the data gaps were available. The mean diurnal variation method was used to fill larger data gaps in meteorological variables. When larger gaps occurred, daytime (NEP) and nighttime (RE at night, RE_night_) carbon fluxes were calculated using the Michaelis–Menten equation (Falge et al., 2001) and the equation suggested by Reichstein et al. (2002), respectively. The equation of Reichstein et al. (2002) used daytime SWC and soil temperature to estimate daytime RE (RE_daytime_). The RE is the sum of RE_daytime_ and RE_night_, and GEP is calculated using NEP and RE.

### 2.5 Uncertainty analysis of carbon fluxes

There are three uncertainty categories of half‐hour averaged carbon fluxes: (a) Random errors caused by statistical and measurement equipment uncertainties, (b) selective systematic uncertainties caused by lack of nocturnal mixing, and (c) systematic uncertainties associated with longer gap filling (Aurela, Laurila, & Tuovinen, 2002; Tang et al., 2016). The growth season of grass species (May–September) was used for further analysis; thus, three uncertainties were calculated during this period. The random error (*E*_r_) calculated through the modeled (*F*_mod_) and observed (*F*_obs_) half‐hour fluxes: $E_{r}=\sqrt{\sum_{i=1}^{n}{\left(F_{\mathrm{obs}}-F_{\mathrm{mod}}\right)}^{2}/\left(n\left(n-1\right)\right)}$, where *n* is the half‐hour carbon fluxes number. The selective systematic uncertainties were mainly caused by the selection of the u* limit (Aurela et al., 2002; Tang et al., 2016). The u* of 0.10, 0.21, and 0.30 m/s was used to test the uncertainties of nighttime carbon fluxes. In addition, as indicated by Aurela et al. (2002) and Tang et al. (2016), the systematic uncertainties caused by gap filling method were estimated—the 3‐day forward and backward environmental parameters were used to fill carbon flux gaps (>3 day) in the equations. Then, the error accumulation principle method was used to combine these errors or uncertainties as suggested by Flanagan and Johnson (2005). The uncertainty assessment in each year was expressed as the percentage relative to carbon flux (Table 1). The averaged uncertainties during 2013–2015 were 7.88 ± 1.34% (GEP), 12.19 ± 1.6% (NEP), and 13.06 ± 0.48% (RE), and smaller than the corresponding carbon flux of a subarctic fen ecosystem (Aurela et al., 2002) and a subtropical coniferous forest ecosystem (Tang et al., 2016).

**Table 1:** The daily average (mean ± *SD*) and coefficients of variation (CVs, %) of PAR (mol/m^2^), SWC_5cm and SWC_20cm (m^3^/m^3^), GEP, NEP, and RE (g C m^−2^ day^−1^) (estimates of uncertainty in %) during May–September of 2013–2015

	SWC_5cm	SWC_20cm	GEP	NEP	RE
2013					
Mean ± *SD*	0.13 ± 0.03	0.15 ± 0.03	1.98 ± 0.63 (6.38%)	0.76 ± 0.40 (10.35%)	1.21 ± 0.47 (12.89%)
CV	22.51	21.39	32.0	52.59	38.7
2014					
Mean ± *SD*	0.14 ± 0.04	0.15 ± 0.04	2.03 ± 0.73 (8.97%)	0.78 ± 0.43 (13.18%)	1.24 ± 0.60 (12.69%)
CV	26. 47	23.62	35.97	55.18	48.3
2015					
Mean ± *SD*	0.12 ± 0.03	0.14 ± 0.03	2.27 ± 0.67 (8.28%)	0.90 ± 0.44 (13.04%)	1.38 ± 0.59 (13.6%)
CV	20.81	18.18	29.6	49	41

CV is the ratio of standard deviation to mean value × 100%. Uncertainty is the percentage (%) relative to carbon flux.

### 2.6 The rainfall pulses response of carbon fluxes and threshold‐delay model

To avoid the effect of vegetation growth on carbon fluxes, the relative response to rainfall of carbon fluxes (*y*_relative_) was calculated by comparing the differences in GEP, NEP, or RE on the day that the maximum value occurred after a specific rainfall event with the value before rainfall.

(1) $$y_{\mathrm{relative}}=\left(y_{\max}-y_{t-1}\right)/y_{t-1}$$

where *y*_max_ is the maximum value of GEP, NEP, or RE after the specific rainfall event and *y*_*t*−1_ is the corresponding value before the rainfall event.

In addition, data selection for rainfall pulse analysis and subsequent threshold‐delay model follows three situations: (a) to avoid the uncertainties of carbon fluxes mentioned in Section 3.3, only daily fluxes with data gaps <2 days were selected; (b) to ensure effectiveness of rainfall pulse, the rainfall lasted >5 days was not used (Jian et al., 2016); (c) 5 days after rainfall event was selected to detect the maximum GEP, NEP, or RE, because previous studies suggested that the longest time that rainfall pulse affect the carbon or water fluxes for grass ecosystem was <5 days (Cheng et al., 2006; Hao et al., 2010; Potts et al., 2006); therefore, interpulse period <6 days was also not used. In the study period of 2013–2015, 21 rainfall events were suitable for analysis and accounted for approximately 41.2% of all rain events.

The threshold‐delay model, calculated through plant physiological responses to rainfall pulses, incorporates the precipitation thresholds and the time delay of plant responses (Ogle & Reynolds, 2004; Zeppel et al., 2008):

(2) $$y_{t}=ky_{t-1}+\delta_{t}$$

(3) $$\delta_{t}=\min\left[y_{\max}\left(1-k\right),\;\delta_{t}^{*}\left(1-y_{t-1}/y_{\max}\right)\right]$$

(4) $$\delta_{t}^{*}=\left\{\begin{array}{cc} \left(\delta_{\max}/\left(R^{U}-R^{L}\right)\right)\left(R_{t-\tau }-R^{L}\right) & R^{L}<R_{t-\tau }<R^{U}\\ 0 & R_{t-\tau }\leq R^{L}\\ \delta_{\max} & R_{t-\tau }\geq R^{U} \end{array}\right.$$

where y_*t*_ is the carbon flux at time *t*;* y*_*t*−1_ is the antecedent carbon flux; *y*_max_ is the maximum value of carbon flux during the studied period; δ_*t*_, δ^*^_t_, and δ_max_ are the actual, potential, and maximum response carbon fluxes, respectively; *R* ^*U*^ and *R* ^*L*^ are the upper and lower threshold of rainfall, respectively; *R*_*t−*τ_ is the effective rainfall amount; τ is the number of days that carbon fluxes take to reach the maximum value after *R*_*t−*τ_; and *k* is the reduction rate.

The *R* ^*L*^ is the lowest rainfall amount that significantly increased carbon fluxes, and the *R* ^*U*^ is the largest rainfall amount such that any greater rainfall amount could not increase additional carbon fluxes (Ogle & Reynolds, 2004). After testing for normal distribution and homogeneity of variance, *R* ^*U*^ and *R* ^*L*^ were identified using an independent sample *t* test between the data before rainfall and the maximum value after a specific rainfall event based on the half‐hour data. The remaining parameters were calculated using daily data. The τ was determined for each rainfall events; *k* and $ \delta_{t}^{*} $ were calculated using Equations (2) and (4) based on 21 rainfall events, respectively. Then, δ_*t*_ was calculated using the Equation (3). The parameters *R* ^*U*^, *R* ^*L*^, and average τ are shown in Table 2 and the other parameters in Supporting Information Table S1.

**Table 2:** Parameters of the threshold‐delay model for rainfall response of GEP, NEP, and RE

Parameter	*R* ^*L*^	*R* ^*U*^	τ
GEP	6.6	21.4	1.67 ± 0.58
NEP	8.5	21.4	2.05 ± 0.67
RE	4.5	16.8	1.1 ± 0.3

*R* ^*L*^ is the lower rainfall threshold (mm), *R* ^*U*^ is the upper threshold (mm), and τ is the time lag (mean ± *SD*, day).

### 2.7 Statistical analyses

Significant differences in *y*_relative_ among rainfall classes (0–5, 50–10, 10–20, 20–30, and >30 mm) were detected through analysis of variance (ANOVA) and Tukey's HSD test (after normal distribution and homogeneity of variance test). Meanwhile, a significant difference in *y*_relative_ among GEP, NEP, and RE at the same rainfall class was also detected through ANOVA and Tukey's HSD test. The fine root surface area of dominant grasses at soil depth 0–20 cm accounted for 87.32% of that for the soil profile 0–50 cm (Supporting Information Figure S1). Therefore, for each of the 21 rainfall events, environmental variables such as rainfall amount (P), SWC_antecedent at 5 cm, and SWC_antecedent at 20 m were selected as factors affecting rainfall responses of GEP, NEP, and RE. To determine which variables most influenced the relative carbon fluxes in response to a rainfall event, firstly, the variables that significantly affected the relative GEP, NEP, and RE were identified through multiple linear regression analysis (stepwise method). Then, the unique contribution of each environmental variable to relative fluxes was identified based on the partial slope and partial determination coefficient (*R* ^2^) value using multiple linear regression analysis, by holding other environmental variables constant (Hair, Black, Babin, Anderson, & Tatham, 2006; Zeppel et al., 2008).

## 3 RESULTS

### 3.1 Variation in environmental factors

**Figure 1:**
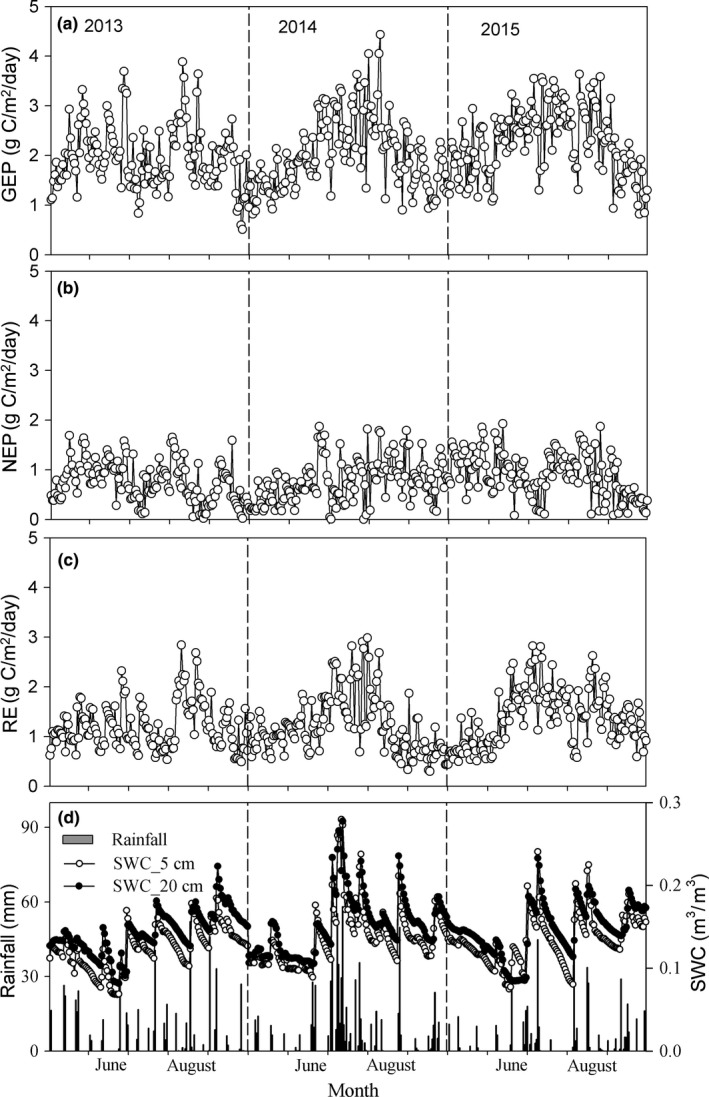
Daily variations of (a) GEP (g C m^−2^ day^−1^), (b) NEP (g C m^−2^ day^−1^), and (c) RE (g C m^−2^ day^−1^), and (d) rainfall amount and soil moistures at 5 and 20 cm depths (SWC_5cm and SWC_20cm, respectively) for May–September during 2013–2015

**Figure 2:**
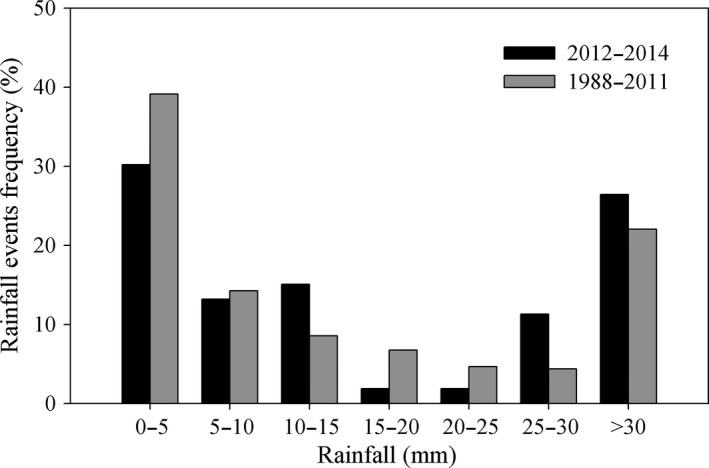
Frequency distribution of rainfall events for May–September during 2013–2015 and 1988–2012

The rainfall amount during May–September 2014 (633.8 mm) was the highest since 1988 and was 1.53 times that during 1998–2012 (414.2 mm). The amounts during the observation period in 2013 (463.7 mm) and 2015 (433.4 mm) were similar to the average value (Figure 1). The rainfall distribution during 2013–2015 was similar to that for 1988–2012, characterized by more frequent small (0–5 mm) and large (>30 mm) events (Figure 2).

The variation of soil moisture at 5 and 20 cm depths (SWC_5cm and SWC_20cm, respectively) was closely related to rainfall events; SWC generally increased after rainfall and then gradually decreased (Figure 1). Consistent with rainfall amounts, the highest average SWC_5cm and SWC_20cm during May–September were in 2014, and the smallest corresponding values were in 2015 (Table 1). The SWC_20cm was generally higher than SWC_5cm during the observation period in the 3 years. In addition, the CVs of SWC_5cm during May–September during 2013–2015 were higher than for corresponding SWC_20cm, suggesting that surface soil water content fluctuated more with rainfall and magnitudes decreased with increasing depth.

### 3.2 Daily variation of carbon fluxes and responses to rainfall

**Figure 3:**
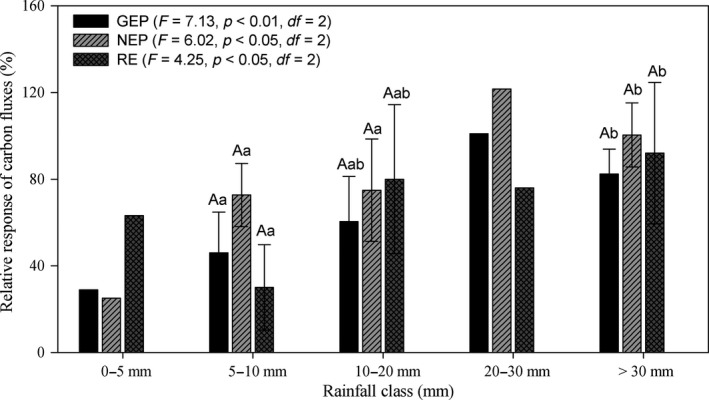
The relative response of daily carbon fluxes to rainfall class (mean ± *SD*, %). The rainfall classes of 0–5 and 20–30 mm were not used for these significant test, because only one effective rainfall amount (4.5 mm) and two effective rainfall amounts (21.8 and 27 mm) were observed during the study period. Different small letters indicate significant differences between rainfall classes for the same carbon fluxes component at *P *< 0.05, and different capital letters indicate significant differences among GEP, NEP, and RE at the same rainfall class at *P *< 0.05. Relative response to rainfall of GEP, NEP, and RE in this Figure was calculated from Equation (1)

The daily variation of GEP, NEP, and RE fluctuated with rainfall events (Figure 1). The highest daily averaged GEP, NEP, and RE during May–September were in 2015, and the smallest corresponding values in 2013 (Table 1). During 2013–2015, the CV of GEP was the smallest, and the CV of NEP was the largest of the three carbon fluxes. Generally, the relative responses of GEP, NEP, and RE significantly differed (*P *< 0.05, *df *= 2) among the three rainfall classes (Figure 3), when 0–5 and 20–30 mm classes were not considered. In addition, no significant difference was observed among GEP, NEP, and RE at specific rainfall classes (Figure 3), when 0–5 and 20–30 mm classes were not considered. The relative response of NEP was largest, with average response as high as 84.24 ± 26.24%, followed by RE (76.11 ± 34.52%) and GEP (68.63 ± 23.73%).

**Figure 4:**
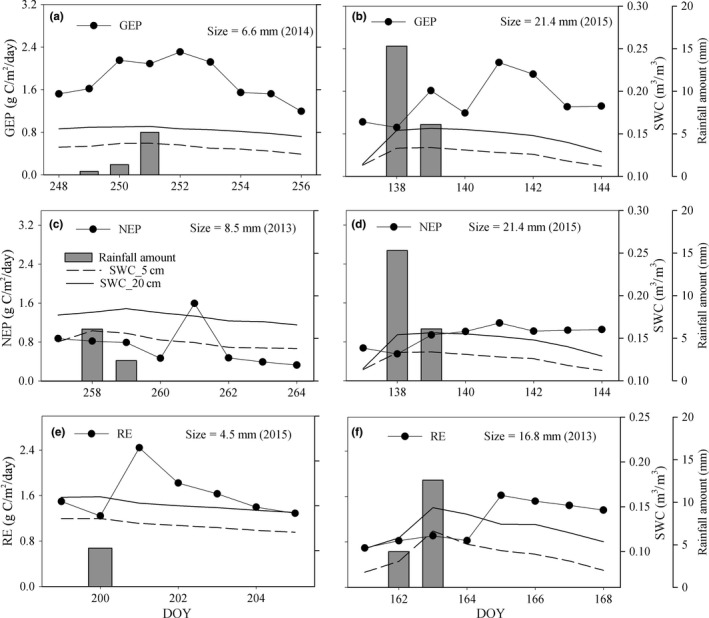
Daily (a and b) GEP, (c and d) NEP, and (e and f) RE after different rainfall events during 2013–2015, and corresponding SWC_5cm and SWC_20cm values

According to the threshold‐delay model, the *R* ^*L*^ for GEP was 6.6 mm (September 2014) (Table 2), and the GEP after rainfall increased by 51.9% compared with the value before rainfall (2.31 and 1.52 g C m^−2^ day^−1^, respectively) (Figure 4). The *R* ^*L*^ for NEP appeared was 8.5 mm (September 2013), and the NEP after rainfall increased by 82.76% compared with the value before rainfall (1.59 and 0.87 g C m^−2^ day^−1^, respectively). In addition, the *R* ^*U*^ for GEP and NEP were both 21.4 mm (May 2014), and the GEP (2.06 g C m^−2^ day^−1^) and NEP after rainfall (1.35 g C m^−2^ day^−1^) increased by 100.97% and 121.6% compared with values before rainfall (1.02 and 0.61 g C m^−2^ day^−1^), respectively. The *R* ^*L*^ and *R* ^*U*^ for RE were 4.5 mm (July 2015) and 16.8 mm (June 2013), respectively, and the corresponding RE after rainfall (2.44 and 1.62 g C m^−2^ day^−1^) increased by 63.2% and 133.9% compared with values before rainfall (Figure 4). The average lag times for GEP, NEP, and RE were 1.67 ± 0.58, 2.05 ± 0.67, and 1.1 ± 0.3 days, respectively (Table 2).

### 3.3 Effect of environmental factors on rainfall responses of carbon fluxes

**Figure 5:**
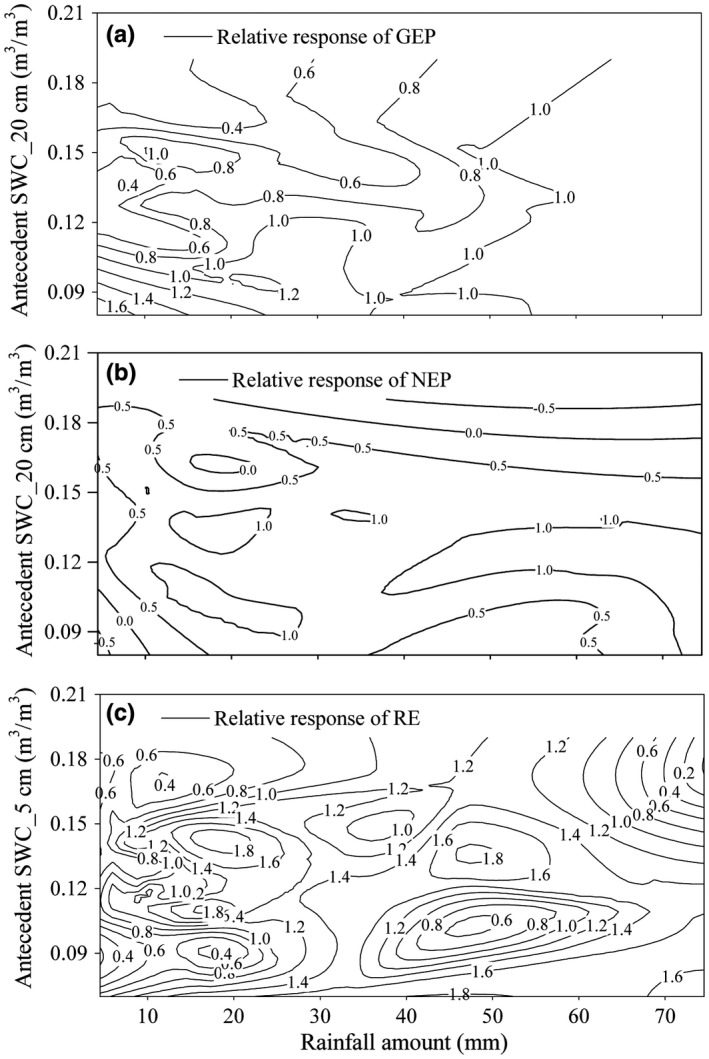
Contour plots of the relative responses (%) of (a) GEP, (b) NEP, and (c) RE following rainfall amount (mm) and antecedent SWC (m^3^/m^3^). Relative response to rainfall of GEP, NEP, and RE in this Figure was calculated from Equation (1)

The rainfall amount and antecedent SWC_5cm was the most influential factor on responses of NEP and RE, respectively, with corresponding partial slopes of 0.46 and −0.52 (Table 3). The partial slope between antecedent SWC_20cm and the relative responses of GEP was −0.50 and was 0.495 between rainfall amount and the relative responses of GEP. The negative partial slopes of antecedent SWC_5cm for RE and NEP, and antecedent SWC_20cm for GEP, suggested that high antecedent soil moisture offset the magnitude of responses of these two fluxes to rainfall (Table 3, Figure 5). Under better soil moisture conditions, smaller increases in GEP and RE occurred even following large rainfall pulses. For example, although 34.5 mm of rainfall occurred during 28–30 July 2013, relative increases in GEP, NEP, and RE were only 55.0%, 56.88%, and 35.45%, respectively, as the corresponding antecedent SWC_20cm and antecedent SWC_5cm were as high as 0.16 and 0.14 m^3^/m^3^. In contrast, the GEP, NEP, and RE increased by 100.97%, 121.6%, and 76.0%, respectively, in response to 21.4 mm of rainfall during 18–19 May 2014, and corresponding antecedent SWC_20cm and antecedent SWC_5cm were both 0.11 m^3^/m^3^.

**Table 3:** Multiple linear regression analysis for relative response to rainfall of GEP, NEP, and RE

	Relative response of GEP		Relative response of NEP		Relative response of RE	
	Partial slope	Partial *R* ^2^	Partial slope	Partial *R* ^2^	Partial slope	Partial *R* ^2^
*P*	0.495	0.38	0.46	0.28	0.36	0.20
Antecedent SWC_5cm	–	–	−0.40	0.23	−0.52	0.34
Antecedent SWC_20cm	−0.50	0.39	–	–	–	–

Antecedent SWC_5cm and antecedent SWC_20cm are antecedent soil moistures at 5 and 20 cm before rainfall, respectively; *P* is rainfall amount; relative responses to rainfall of GEP, NEP, and RE in this Table were calculated from Equation (1). “–” indicates that the partial slope did not pass the significance test.

## 4 DISCUSSIONS

### 4.1 Rainfall pulse effect on carbon fluxes

Positive relationships between rainfall amount and carbon fluxes have been observed in many grass ecosystems (Chen et al., 2015; Reichstein et al., 2005). However, total rainfall amount is not always an accurate indicator of the water available to affect carbon cycling (Hao et al., 2013; Scott et al., 2010). Similar to studies in a temperate steppe (Hao et al., 2013) and a tallgrass prairie ecosystem (Harper, Blair, Fay, Knapp, & Carlisle, 2005), rainfall amount did not significantly influence monthly GEP (*R* ^2^ = 0.17, *P* = 0.13, *n* = 15), NEP (*R* ^2^ = 0.01, *P* = 0.74, *n* = 15), or RE (*R* ^2^ = 0.22, *P* = 0.07, *n* = 15) in the present study. Tang et al. (2016) and Liu et al. (2017) suggested that carbon flux component responses to precipitation may vary depending on nonlinear responses of plants and soil microbes. Plant physiological response is expected only when the rainfall amount promotes soil water status. Previous studies indicated that a small rainfall amount (e.g., 5 mm) was the lower threshold to stimulate plant activities (Tang et al., 2018; Zeppel et al., 2008;; Zhao & Liu, 2010). However, Thomey et al. (2011) indicated that small rainfall events may only increase microbial respiration in shallow soil layers and could not trigger plan root activities in relative deeper soil layers. In the current study, SWC_5 cm but not SWC_20 cm was affected by rainfall of 4.5 mm; therefore, RE rather than GEP or NEP significantly increased (Figure 4e). In addition, enhanced GEP and NEP occurred in response to larger rainfall events by recharging deeper soil in this semiarid grass ecosystem (Figure 4a and c). Potts et al. (2006) suggested that only heavy rainfall improved plant photosynthetic capacity and increased carbon absorption rate, and the response time between rainfall and carbon fluxes may be mainly determined by plant root distribution. For example, Hao et al. (2010) indicated that 3 and 5 mm were the threshold rainfall amounts that significantly affected RE and NEP in a temperate steppe ecosystem, respectively, and the corresponding time lags after rainfall were 1–2 and 3–4 days. In the present study, 58.2% of fine root surface area for the dominant grasses was an indicator of soil water uptake and was mainly distributed in the 10‐ to 20‐cm soil layer (Supporting Information Figure S1). Therefore, compared with RE, there were longer time lags for GEP and NEP after rainfall (Table 2, Figure 4e).

Although rainfall pulses may enhance carbon fluxes by recharging soil water in semiarid regions, there is little variation and may even decrease carbon fluxes after heavy rainfall (Hao et al., 2013; Polley, Frank, Sanabria, & Phillips, 2008). The *R* ^*U*^ that stimulated GEP and NEP was 21.4 mm, which was slightly higher than that for RE (16.8 mm) (Table 2). Austin et al. (2004) and Huxman et al. (2004) suggested that large rainfall may reduce the pool of soil available nitrogen, limiting soil microbe activities, and thereby constraining RE. In addition, with the increase in SWC, plant root dysfunction may be induced by declining oxygen diffusion, thus inhibiting microbial activity and decreasing plant photosynthesis (Hao et al., 2017; Huxman et al., 2004). Furthermore, Pan and Shangguan (2005) suggested that no runoff was observed in silt loam in Loess Plateau when grass coverage was approximately 90%, under slope of 15° and rainfall intensity of 1.5 mm/min. In a plant cover system in Loess Plateau, with similar bulk density to that in the present study (1.36 ± 0.11 g/cm^3^), no runoff occurred when rainfall intensity reached to 0.74 mm/min (Pan et al., 2017). However, because no direct assessment was conducted in the present study, the possibility runoff following large amounts of rainfall should be considered when analyzing the pulse effect of large rainfall amount on carbon fluxes in further research in this region.

### 4.2 Antecedent SWC influences the rainfall response of carbon fluxes

Some models assume that individual rainfall pulses act independently; however, the rainfall sensitivity of carbon fluxes may depend on antecedent soil water (Hao et al., 2017; Huxman et al., 2004; Zeppel et al., 2008). Xu and Baldocchi (2004) and Potts et al. (2006) indicated that rainfall pulses may strongly stimulate carbon fluxes in low soil moisture conditions. In the current study, higher soil moisture offset the positive effect of rainfall relative response of GEP, NEP, and RE, with partial regression coefficients of −0.50, −0.40, and −0.52, respectively (Table 3, Figure 5). Wiegand, Snyman, Kellner, and Paruelo (2004) suggested that the antecedent soil water effect on photosynthesis capacity in grassland mainly depended on the deep roots of grass species. In the studied semiarid grass ecosystem, the negative effect of SWC_20 cm on GEP suggested that deep plant root mainly affects the plant photosynthesis activities. The negative effect of antecedent SWC_5cm on RE (Table 3) may be due to the effect of shallow soil water on heterotrophic respiration (soil microbial activity) and on autotrophic respiration of the shallow fine roots of *G. uralensis*. Meanwhile, the antecedent SWC_5cm was also negatively affected the balance between GEP and RE, which may partially attribute to the large proportion of GEP that RE represents (>61%). The result also indicated that the effect of carbon lose on carbon sequestration capacity should be considered even in grass growth period (Guo et al., 2016). However, no effect of antecedent soil water in controlling the response of plants to rainfall was observed in Chihuahuan desert (Reynolds, Kemp, Ogle, & Fernandez, 2004). Hao et al. (2013) and Reynolds et al. (2004) attributed less impact of antecedent soil water to strong soil evaporation in desert, because there is generally low water availability during rainless periods.

### 4.3 Implications of rainfall pulse response of carbon fluxes

Small (<5 mm) and large (>30 mm) rainfall events dominated the rainfall distribution during the growing seasons of the past 35 years (Figure 2). Although only one 4.5 mm was selected within the 0–5 mm rainfall class (Figure 3), we predict that more frequent events of 0–5 mm will enhance RE based on the *R* ^*L*^ for these carbon fluxes in Table 2. In addition, large rainfall pulses may enhance soil moisture storage, especially for deeper soil layers, and subsequent small rainfall pulses may enhance grass GEP in the present study. Although rainfall amount was positively (*P* < 0.05) affect the relative response to rainfall of GEP, NEP, and RE (Table 3), no significant relationship was observed when rainfall amount was selected larger than *R* ^*U*^ for each carbon fluxes (Supporting Information Figure S2). Therefore, constrained carbon fluxes in response to heavy rainfall pulses should also be considered when successive large rainfall pulses occur.

In addition, most fine roots of *G. uralensis* were distributed in shallow soil depth (0–10 cm) than those of *A. scoparia* (10–20 cm) (Supporting Information Figure S1). Although the water sources of carbon fluxes in response to rainfall pulses were not directly measured in this study, the distribution of fine roots is considered a valid indicator of water sources in many grass ecosystems (Silvertown, Araya, & Gowing, 2015; Yang, Auerswald, Bai, & Han, 2011). In this semiarid region, we predict that more frequent rainfall events of 0–5 mm will benefit *G. uralensis*, and more frequent >30 mm events will favor *A. scoparia*. We also predict that the composition of these grass species and rainfall pulse sensitivity of carbon fluxes for the studied grass ecosystem may alter following shifts in rainfall distribution, even if total rainfall remains stable.

## 5 CONCLUSIONS

Rainfall pulse sensitivity of GEP, NEP, and RE was assessed in a temperate grass ecosystem in a semiarid region, and the unique contributions of environmental variables to these carbon fluxes were determined. The NEP was more sensitive to rainfall pulses and RE was more sensitive to SWC_antecedent, based on the partial slope analysis. In addition, SWC_antecedent significantly offset the positive effect of rainfall amount on the response of GEP, NEP, and RE to rainfall pulses. Moreover, carbon fluxes may also alter in response to changed rainfall distributions, even if total rainfall remains stable. Further investigations of water sources, as well as rainfall characteristics, are needed for selected grass species in terms of carbon sequestration in this semiarid region.

## CONFLICT OF INTEREST

We declare that we have no conflict of interest.

## AUTHORS CONTRIBUTION

YKT, JJ, and YMC conceived and designed the research; YKT, JJ, and CC conducted the experiments; YKT and XW designed and conducted the analyses and interpretation; YKT, XW, and YMC wrote the manuscript.

## DATA ACCESSIBILITY

Carbon fluxes, rainfall, and soil water content, as well as the root surface area of *A. scoparia* and *G. uralensis*, are available through Dryad (https://doi.org/10.5061/dryad.4fq5vr6).
