# Supplementary material for: Rainfall pulse response of carbon fluxes in a temperate grass ecosystem in the semiarid Loess Plateau
Source: Ecol Evol. 2018 Oct 23;8(22):11179–89. doi: 10.1002/ece3.4587 (PMC6262730; doi:10.1002/ece3.4587)
Supplement: Supplementary file 3 [file ECE3-8-11179-s003.docx]

**Table S1** The reduction rate (*k*), maximum value of carbon flux during the studied period (*y_max_*, g C m^-2^ day^-1^), average (mean±SD) *δ^*^_t_* potential response carbon fluxes (*δ^*^_t_*, g C m^-2^ day^-1^), and average actual response carbon fluxes (***δ****_t_*, g C m^-2^ day^-1^) of the threshold-delay model for rainfall response of GEP, NEP and RE.

|  | *k* | *y_max_* | *δ^*^_t_* | *δ_t_* |
| --- | --- | --- | --- | --- |
| GEP | 0.73 | 4.43 | 2.65±1.49 | 0.77±0.45 |
| NEP | 0.5032 | 1.93 | 1.47±1.02 | 0.40±0.37 |
| RE | 0.533 | 2.99 | 1.00±0.61 | 0.85±0.43 |





**Figure S1** Vertical distribution of root surface area (m^2^) of *A. scoparia* and *G. uralensis*.





**Figure S2** Relationship between rainfall amount and relative response of (a) GEP, (b) NEP and (c) RE. Rainfall amount was separated into <=21.4 mm and > 21.4 mm for GEP and NEP, and <=16.8 mm and > 16.8 mm for RE. Relative response to rainfall of GEP, NEP and RE in this Figure was calculated from Equation (1).
